# News Items

**Published:** 1917-01

**Authors:** 


### News Items.

Dr. C. M. Payne, formerly of Corpus Christi, is now located at Benford, Texas.

---

Dr. T. H. Harrell, of Gonzales, has just returned from a two months' trip to Chicago and Rochester, where he has been doing post-graduate work.

---

Owing to a severe wrist injury, Dr. F. S. White, of San Antonio, has been unable to continue for the present his series of articles on incipient insanity. We hope, however, to have another article from his pen for the next issue.

---

The Southern Gastro-Enterological Association was organized in Atlanta on November 15, 1916.

Active membership in this society will be limited to those investigators and practitioners of the seventeen Southern States who are engaged primarily in the diagnosis and treatment of diseases of the digestive system.

Regular meetings will be held annually, the next place of meeting yet to be announced.

The following officers were elected: Dr. J. C. Johnson, Atlanta, President; Dr. J. T. Rogers, Savannah, Vice-President; Dr. Marvin H. Smith, Jacksonville, Secretary-Treasurer.

Councilors: Dr. S. K. Simon, New Orleans; Dr. G. M. Niles, Atlanta, and Dr. Seale Harris, Birmingham.

Admission and Ethics: Dr. Geo. C. Mizell, Atlanta; Dr. J. E. Knighton, Shreveport, and Dr. J. B. Fitts, Atlanta.

---

### Do Doctors Make Their Voices Heard Enough?

BY DR. INGE, DEAN OF ST. PAUL'S CATHEDRAL, LONDON.

The doctors have much to teach us. I am going to urge very respectfully in the course of this sermon that they do no make their voices heard quite enough. In their private practice they have to a large extent succeeded to some of the functions of the medieval priests. It is they who now hear the confessions of anxious and conscience-stricken penitents; it is they who prescribe dietary disciplines and various quaint penances; it is they who send people on pilgrimages to distant lands.

Moreover, owing to the state of neglect into which the art of spiritual therapeutics has fallen in Protestant countries, the physician usually knows more than the clergyman about the real springs of action, the secret causes of sin and sorrow, the subtle and delicate influences by which soul and body affect each other, the mysterious and melancholy trammels of morbid heredity, and the unrecognized heroism of struggles against it. To him, and not always to the clergyman, are known the storms and stress which often accompany the beginning and sometimes the end of life; the nervous instability which may show itself in mental depression, in foolish and violent partizanship, in loss of natural affection, and in many other obscure ways. In short, when a physician of the soul preaches to physicians of the body he is influencing public opinion in those matters wherein its members alone can speak with authority?—*Selected.*

